# The association between meat and fish consumption and bladder cancer risk: a pooled analysis of 11 cohort studies

**DOI:** 10.1007/s10654-021-00762-4

**Published:** 2021-05-25

**Authors:** Mostafa Dianatinasab, Anke Wesselius, Tessa de Loeij, Amin Salehi-Abargouei, Evan Y. W. Yu, Mohammad Fararouei, Maree Brinkman, Piet van den Brandt, Emily White, Elisabete Weiderpass, Florence Le Calvez-Kelm, Marc J. Gunter, Inge Huybrechts, Fredrik Liedberg, Guri Skeie, Anne Tjonneland, Elio Riboli, Maurice P. Zeegers

**Affiliations:** 1 Department of Complex Genetics and Epidemiology, School of Nutrition and Translational Research in Metabolism, Maastricht University, Universiteitssingel40 (RoomC5.570), 6229 ER, Maastricht, The Netherlands; 2 Nutrition and Food Security Research Center, Department of Nutrition, School of Public Health, Shahid Sadoughi University of Medical Sciences, Yazd, Iran; 3 Department of Epidemiology, Shiraz University of Medical Sciences, Shiraz, Iran; 4 Department of Clinical Studies and Nutritional Epidemiology, Nutrition Biomed Research Institute, Melbourne, Australia; 5 Cancer Epidemiology Division, Cancer Council Victoria, 615 St Kilda Road, Melbourne, VIC 3004 Australia; 6 Department of Epidemiology, Schools for Oncology and Developmental Biology and Public Health and Primary Care, Maastricht University Medical Centre, Maastricht, The Netherlands; 7 Fred Hutchinson Cancer Research Center, Seattle, WA USA; 8 International Agency for Research on Cancer World Health Organization, Lyon, France; 9 Department of Urology, Skåne University Hospital, Malmö, Sweden; 10 Institution of Translational Medicine, Lund University, Malmö, Sweden; 11 Department of Community Medicine, UIT The Arctic University of Norway, Tromsø, Norway; 12 Danish Cancer Society Research Center, Copenhagen, Denmark; 13 Department of Public Health, University of Copenhagen, Copenhagen, Denmark; 14 Department of Epidemiology and Biostatistics, School of Public Health, Imperial College London, London, UK; 15 CAPHRI School for Public Health and Primary Care, Maastricht University, Maastricht, The Netherlands; 16 School of Cancer Sciences, University of Birmingham, Birmingham, UK

**Keywords:** Bladder cancer, Meat, Fish, Risk factor, Epidemiology

## Abstract

Evidence on the effects of meat consumption from different sources on the risk of bladder cancer (BC) is limited and controversial. Therefore, this study aimed to evaluate the associations between meat consumption and BC risk using a pooled data approach. Individual data from 11 prospective cohorts comprising 2848 BC cases and 515,697 non-cases with a total of 5,498,025 person-years of follow-up was pooled and analysed to investigate the potential associations between total red meat and products, red meat, processed meat, poultry and total fish and BC risk. Hazard ratios (HRs), with corresponding 95% confidence intervals (CIs), were estimated using Cox regression models stratified on cohort. Overall, an increased BC risk was found for high intake of organ meat (HR comparing highest with lowest tertile: 1.18, 95% CI: 1.03, 1.36, p-trend = 0.03). On the contrary, a marginally inverse association was observed for total fish intake and BC risk among men (HR comparing highest with lowest tertile: 0.79, 95% CI 0.65, 0.97, p-trend = 0.04). No associations were observed for other meat sources. Results of this prospective study suggest that organ meat consumption may be associated with BC development. Replication in large-scale prospective studies and investigation of possible causal mechanisms is needed.

**Supplementary Information:**

The online version contains supplementary material available at 10.1007/s10654-021-00762-4.

## Introduction

Cancer of the bladder (BC) is among the top ten most common cancer types in the world, with approximately 573,000 new cases and 213,000 deaths [1]. Incidence rates of BC are the highest in Southern and Eastern Europe Africa and the Middle East, and in North America [2]. BC occurs mainly in men and elderly [1] and approximately 75% of the bladder cancers are non-muscle-invasive (NMIBC) which require intensive treatment and follow-up measures, thereby posing a large burden on national health care budgets [3]*.* Epidemiological studies have identified several factors which potentially influence BC risk, including; sex, smoking, age and certain occupations [3, 4]. Well-established BC carcinogens include aromatic amines like heterocyclic amines (HCAs), polycyclic aromatic hydrocarbons (PAHs) and arsenic and repetitive urinary tract infections have also been reported to increase BC risk [5]. In addition, a wider range of evidence is becoming available on the plausible role of dietary factors in BC occurrence [5]. However, the latest World Cancer Research Fund International (WCRF) report stated that evidence from epidemiologic studies on the association between diet and BC is still scarce and largely inconsistent [6].

Meat is a rich source of multiple potentially carcinogenic compounds, including nitrate, nitrite, HCAs and PAHs, with a known effect on tumor growth induction [7–10]. Since these compounds are excreted in the urine and therefore come in close contact with the inner lining of the bladder wall, these components might play an important role in BC development [11].

There is however limited and inconsistent epidemiological evidence on the association between meat consumption and BC. While a Swedish cohort study found no association between the consumption of red meat, processed meat, poultry, or fried meats/fish and BC risk [12], other prospective cohort studies suggested an increased BC risk with cumulative consumption of processed red meat [13, 14]. A positive association between meat consumption and BC risk was also confirmed by a meta-analysis, including five cohort and eight case-control studies from all over the world. It was shown that an increment of 50 g of processed meat per day was associated with 20% increased risk of BC [15]. In addition, the authors showed that red meat consumption was associated with BC, with a 51% increased risk per increment of 100 g per day. However, this association with red meat consumption could only be observed among the included case-control studies. A more recent meta-analysis only identified a positive association between red and processed meat among Americans, while an absence of an association was observed for individuals from other continents [16].

These controversial findings might be due to the small sample sizes of previously conducted studies, which consequently would lack statistical power to detect significant associations. Although meta-analysis might overcome this power issue, they solely rely on previously published data, thereby potentially introducing reporting bias. The present study, therefore, aims to provide a more comprehensive estimate for the associations between meat consumption and BC risk, by pooling individual data from 11 cohort studies, thereby not only increasing the power to detect small effect sizes, but also allowing for data homogenization and common adjustment for potential confounding factors.

## Methods

### Study sample

Data were derived from the BLadder cancer Epidemiology and Nutritional Determinants consortium (BLEND) [17]. BLEND is a large international epidemiology consortium, aimed to pool available data from epidemiological studies on diet and BC [17]. BLEND consists of 19 case-control studies and 16 cohort studies. Eleven cohort studies, with a total of 518,545 participants, 2848 of whom developed incident BC, had sufficient information on both meat and fish consumption, and on the most important covariates gender and smoking, to be eligible for inclusion in the present study. These studies originated from 11 countries [i.e. Europe: European Prospective Investigation into Cancer and Nutrition cohort studies (EPIC) [18] (Denmark [19], France [20], Germany [21], Italy [22], The Netherlands [23], Norway [24], Spain [25], Sweden [26, 27], United Kingdom [28, 29]), Netherlands cohort study (NLCS) [30]; and North America: VITamins and Lifestyle cohort study (VITAL) [31]].

### Data collection and pre-processing

Details on the protocol of the BLEND consortium have been described in the BLEND methodology paper [17]. Briefly, the primary data from all the included studies were assembled into an integrated database. Data were checked and the food consumption was converted to grams per day (g/day) by the use of country specific food tables and the frequency responses. National specific standard portions sizes for each food item were used to calculate consumption in g/day. Each study ascertained incident bladder cancer cases, defined to include all subjects with urinary bladder neoplasms according to the International Classification of Diseases for Oncology (ICD-O-3 code C67) using population-based cancer registries, health insurance records, or medical records [32, 33]. Dietary data were obtained using self-administered or trained interviewer administered food frequency questionnaire (FFQ) that was validated on either food groups [31, 34, 35], and/or energy intake [35, 36]. For each study, participants were asked to report on their usual intake during the year before study enrolment of meat and fish. These data were harmonized using the hierarchal Eurocode 2 food coding system developed by the European Union [37], with weekly, monthly or yearly intake converted to grams (g) per day. This resulted in an aggregated dataset with unified dietary intakes across the different studies included.

### Dietary meat consumption

By conducting a comprehensive review of the literature, we were able to use a more common definition of different meat categories [12, 13, 15, 38]. Dietary meat consumptions were categorized in the following groups including total meat and meat products (all meat groups except fish), total red meats and products (total meat and meat products minus poultry), red meat (beef, veal, mutton/lamb and pork), processed meats (preserved meat and meat products), organ meat (liver and other offal), poultry, and fish (fish and fish products). As a result of data availability, red meat, processed meat, organ meat, poultry, and fish consumption were calculated in grams per 1000 kilocalories per day (g/1000 kcal/day, nutrient density method), to account for total energy intake and reduce extraneous variation in dietary intakes [39, 40], and were categorized into tertiles for individual meat types [40]. Then, dietary meat consumptions were divided into 3 groups based on a tertile ordered distribution: low consumption (tertile 1), medium consumption (tertile 2) and high consumption (tertile 3).

### Other variables

In addition to dietary consumption information, other baseline data included study characteristics including study design, method of dietary assessment, recall period of dietary consumption and geographical region, demographic information (age, sex and ethnicity), pathology of BC (non-muscle-invasive bladder cancer [NMIBC] and muscle-invasive bladder cancer [MIBC]), and smoking status (current/former/never) and quantity (packs/year).

### Statistical analyses

Baseline characteristics of the study participants, meat sources and other potential confounders were compared between case and non-case groups using independent samples t-test, for continuous variables, or chi-square for categorical variables. Cox proportional hazard modelling approach was used with age at recruitment as the starting point on the time scale to assess the association between consumptions of meat and BC risk. Hazard ratios (HRs) and 95% confidence intervals (CIs) for developing BC were calculated with the first tertile assigned as reference group. The proportional hazards assumption was examined graphically, and we found no apparent violation to the assumption. Survival time was estimated by subtracting age at exit by age at entry in the cohort as T0, thereby correcting for age in the analysis. Also, study was included as a random effect. The Cox regression models were performed as crude, and based on literature review adjusted model-1 for: age, sex, smoking status (never, former or current smoker), total energy intake in kilocalories, and additionally for: vegetables and fruits consumption (model-2). In addition, when testing for white meat and fish consumption analyses were corrected for red meat intake and vice versa (model 3).

To understand the relevance of plausible effect modification, interaction terms for sex, age and smoking status, and meat- and fish consumption were alternately added to the fully adjusted regression models. This was done by adding the multiplication of meat- and fish consumption in tertiles and: (a) the categorized age (< 40, 40–50, 50–60, > 60), (b) gender, (c) smoking status (current, former and smokers). The Wald-test was used to test for the presence of interaction, and p-interaction < 0.05 was considered statistically significant. Based on the knowledge that BC subtype (i.e. NMIBC and MIBC) have a different etiology, additional subgroup analyses were performed on BC subtypes.

We further assessed the potential dose–response relations of meat consumptions with BC risk by fractional polynomial regression using the ln (natural logarithm) of the HRs (model 3) across categories of consumption, in which the best-fitting second-order fractional polynomial regression model was defined as the model with the lowest deviance [40, 41]. A likelihood ratio test was used to assess the difference between the nonlinear (i.e., the absolute dose and dose squared) and linear (i.e., the absolute dose) models to test for linearity or nonlinearity [41]. For this, we categorized each source meat to six groups including (a) total meat and meat products, (b) red meats, (c) processed meats, (d) organ meats, (e) poultry and (f) fish and fish products into 10 doses (g/1000 kcal/day) according to the range of consumption of meat sources, by which the intervals of each consumption were different. P values for trend were estimated by assigning medians to each category of consumption as a continuous variable.

Finally, in order to determine the single study effect, sensitivity analyses were performed by removing each individual study in turn from the main analysis. All statistical analyses were performed using Stata/SE version 14.2. *P* values less than 0.05 were considered as statistically significant.

## Results

### Baseline characteristics

The baseline characteristics of the study population are shown in Table 1. Baseline characteristics for the 11 included cohort studies individually are shown in Supplementary Table 1. Dietary data from 518,545 study participants, including 2848 incident cases and 515,697 non-cases with a total of 5,498,025 person-years of follow-up (median follow-up: 11.3 years) were analyzed. The study population consisted of 1088 NMIBC cases (63%) and 648 MIBC cases (37%).

**Table 1:** Baseline characteristics meat sources among non-cases and bladder cancer cases in the END international study

Categories of data	Cases	Non-cases	*P* value
	n = 2848	n = 515,697	
*Baseline age year (mean (SD))*	60.6 (7.28)	52.5 (10.09)	< 0.001^
*Person-year*	Total: 21,210.08	Total: 5,476,815	< 0.001^
	Median: 7.45	Median: 10.62	
*Sex n (%)*			< 0.001^
Men	2144 (75.3)	164,953 (32.0)	
Women	704 (24.7)	350,744 (68.0)	
*Smoking status n (%)*			< 0.001*
Current	1118 (39.3)	107,108 (20.8)	
Former	1183 (41.5)	154,474 (30.0)	
Never	547 (19.2)	254,115 (49.2)	
*Dietary meat sources, g/1000 kcal/day (mean (SD))*			
Total meat and meat products	49.06 (28.4)	49.38 (30.65)	0.571^
Total red meats and products	39.98 (26.37)	39.21 (27.40)	0.135^
Red meats	17.38 (17.96)	15.62 (17.15)	< 0.001^
Processed meats	16.34 (13.93)	15.42 (13.08)	< 0.001^
Organ meats	3.11 (4.43)	2.54 (4.53)	< 0.001^
Poultry	8.87 (9.95)	9.99 (11.56)	0.731^
Fish and fish products	3.58 (5.42)	5.76 (6.83)	< 0.001^
*Potential confounders*			
Energy intake, kcal/day (mean (SD)	2179.13 (630.32)	2051.59 (642.12)	< 0.001^
Fruits, g/1000 kcal/day (mean (SD))	77.39 (77.63)	91.74 (222.40)	0.776^
Vegetables, g/1000 kcal/day (mean (SD))	135.88 (103.18)	151.51 (380.96)	< 0.001^
*Ethnicity (%)*			
Caucasian	2834 (99.6)	511,934 (99.3)	0.094*
Non-Caucasian	12 (0.4)	3507 (0.7)	

*SD* standard deviation, *g* gram, *mg* milligram, *ml* milliliters, *kcal* kilocalorie

^Based on independent sample t-test. *Based on Chi-2 test

In total, 167,095 (32%) men and 351,444 (68%) women were included. As shown in Table 1, compared to non-cases, BC cases were more likely to be men (75%) and to be current (39%) or former smokers (41%). Mean (± SD) age for was 60.6 (± 7.3) for cases and 52.5 (± 10.1) for non-cases. The median (interquartile) time from exposure collection to BC diagnosis was 8.5 years (4.9, 12.0). Almost all participants were Caucasian [i.e., 99.6% of the cases and 99.3% of the non-case (*P* = 0.09)].

Regarding dietary factors, compared to non-cases, cases had a higher mean (± SD) consumption of all assessed food items (i.e. total red meat and products 39.9 (26.4) vs. 39.2 (27.4), red meats 17.4 (17.9) vs. 15.6 (17.1), processed meats 16.3 (13.9) vs. 15.4 (13.1), organ meats 3.1 (4.4) vs. 2.5 (4.5), energy intake 2179.1 (630.3) vs. 2051.6 (642.1), except for poultry (8.9 (9.9) vs. 10.0 (11.6)), fish and fish products (3.58 (5.4) vs. 5.7 (6.8)), vegetables (135.9 (103.2) vs. 151.5 (380.9)), and fruits (77.4 (77.6) vs. 91.7 (222.4)), which showed to be consumed in a lower amount among cases (Table 1).

### Associations between meat consumption and BC risk comparing high to low consumption

The results of the Cox regression for subsequent categories of meat consumption are shown in Table 2. We found that greater consumption of organ meats was associated with an increased risk of BC (model 2: HR comparing highest to lowest tertile: 1.18, 95% CI: 1.03, 1.36, p-trend = 0.03). This association remained stable after additional adjustment for poultry meat and fish intake (model 3: HR comparing highest to lowest tertile: 1.21, 95% CI: 1.05, 1.38, p-trend = 0.014). An inverse association between higher consumption of poultry meat and risk of BC was observed (model 2: HR comparing highest to lowest tertile: 0.71, 95% CI: 0.65, 0.78, p-trend < 0.001). However, after adjustment for red meat intake this association disappeared (model 3: HR comparing highest to lowest tertile: 0.98 95% CI: 0.84, 1.12, p-trend 0.54) (Table 2). Furthermore, a marginally non-significant association between total fish and fish products (model 2: HR comparing highest with lowest tertile: 0.84, 95% CI 0.72, 1.00, p-trend = 0.08; model 3: HR comparing highest with lowest tertile: 0.89, 95% CI 0.63, 1.25, p-trend = 0.369) and the risk of BC was observed. No associations were found for any other meat sources.

**Table 2:** Hazard ratio (HR) and 95% confidence interval (CI) of the association of meat and meat types, and risk of BC based on tertiles of meat and meat types

Meat and meat types		Tertile 1	Tertile 2	Tertile 3	P trend
		HR (95%CI)*	HR (95%CI)	HR (95%CI)	
Total red meats and products	Participants	(856/171,991)	(1103/171,741)	(889/171,959)	–
	(cases/non-cases)				
	Person-years	1,790,142	1,845,531	1,862,353	–
	Crude	1 (reference)	1.25 (1.15, 1.36)	1.13 (1.04, 1.23)	0.007
	Model 1^a^	1 (reference)	1.13 (1.04, 1.24)	0.96 (0.87, 1.05)	0.222
	Model 2^b^	1 (reference)	1.12 (1.02, 1.23)	0.94 (0.85, 1.03)	0.085
	Model 3^c†^	1 (reference)	1.08 (0.94, 1.23)	1.01 (0.84, 1.21)	0.248
Red meats	Participants	(445/148,483)	(577/148,350)	(613/148,316)	–
	(cases/non-cases)				
	Person year	1,671,793	1,636,671	1,666,877	–
	Crude	1 (reference)	1.18 (1.04, 1.38)	1.13 (0.99, 1.29)	0.079
	Model 1^a^	1 (reference)	1.08 (0.95, 1.23)	0.99 (0.86, 1.13)	0.750
	Model 2^b^	1 (reference)	1.09 (0.96, 1.24)	1.02 (0.89, 1.17)	0.868
	Model 3^c†^	1 (reference)	1.06 (0.93, 1.20)	1.03 (0.88, 1.21)	0.721
Processed meats	Participants	(505/148,425)	(561/148,365)	(569/148,359)	–
	(cases/non-cases)				
	Person year	1,665,703	1,658,865	1,650,773	–
	Crude	1 (reference)	1.16 (1.04, 1.30)	1.30 (1.16, 1.45)	< 0.001
	Model 1^a^	1 (reference)	0.95 (0.84, 1.07)	0.99 (0.88, 1.11)	0.895
	Model 2^b^	1 (reference)	0.94 (0.84, 1.06)	0.98 (0.88, 1.11)	0.822
	Model 3^c†^	1 (reference)	0.89 (0.72, 1.10)	0.95 (0.73, 1.24)	0.304
Organ meats	Participants	(389/149,366)	(541/147,560)	(705/148,223)	–
	(cases/non-cases)				
	Person year	1,658,709	1,676,021	1,640,611	–
	Crude	1 (reference)	1.31 (1.15, 1.49)	1.48 (1.29, 1.69)	< 0.001
	Model 1^a^	1 (reference)	1.25 (1.09, 1.43)	1.20 (1.05, 1.39)	0.015
	Model 2^b^	1 (reference)	1.21 (1.06, 1.39)	1.18 (1.03, 1.36)	0.032
	Model 3^c†^	1 (reference)	1.22 (1.06, 1.40)	1.21 (1.05, 1.38)	0.014
Poultry	Participants	(1022/171,827)	(977/171,867)	(849/171,997)	–
	(cases/non-cases)				
	Person year	1,891,568	1,858,982	1,747,476	–
	Crude	1 (reference)	0.77 (0.71, 0.84)	0.62 (0.57, 0.68)	< 0.001
	Model 1^a^	1 (reference)	0.82 (0.75, 0.89)	0.73 (0.67, 0.81)	< 0.001
	Model 2^b^	1 (reference)	0.81 (0.74, 0.89)	0.71 (0.65, 0.78)	< 0.001
	Model 3^c‡^	1 (reference)	0.91 (0.81, 1.04)	0.98 (0.84, 1.12)	0.54
Total fish and fish products	Participants	(252/61,489)	(473/61,269)	(812/60,929)	–
	(cases/non-cases)				
	Person year	666,381.6	603,304.3	497,419.5	–
	Crude	1 (reference)	0.73 (0.61, 0.85)	0.54 (0.47, 0.63)	< 0.001
	Model 1^a^	1 (reference)	0.85 (0.73, 0.96)	0.88 (0.75, 1.03)	0.257
	Model 2^b^	1 (reference)	0.85 (0.73, 1.00)	0.84 (0.72, 1.00)	0.080
	Model 3^c‡^	1 (reference)	0.92 (0.76, 1.11)	0.89 (0.63, 1.25)	0.369

*HR hazard ratio, *CI* confidence interval

^a^Adjusted for age, sex, smoking status and total energy intake

^**b**^Adjusted for model 1 + vegetables and fruits intakes

^c^Adjusted for †model 2 + poultry and fish intake ‡model 2 + red meat intake

### Subgroup analysis

A significant interaction was observed between fish consumption and gender and smoking (p-interaction = 0.03, 0.01, respectively). No other interaction terms showed to be relevant.

An inverse association between total fish and fish products consumption and BC risk in men (model 2: HR comparing highest with lowest tertile: 0.81, 95% CI: 0.67, 0.98, p-trend = 0.03; model 3: HR comparing highest with lowest tertile: 0.79, 95% CI: 0.65, 0.97, p-trend = 0.04) was observed, but no association was found in women (model 2: HR comparing highest with lowest tertile: 0.96, 95% CI: 0.63, 1.45, p-trend = 0.69; and model 3: HR comparing highest with lowest tertile: 1.07, 95% CI: 0.76, 1.51, p-trend = 0.658, p-heterogeneity = 0.02) (Table 3). No significant association for fish intake and BC risk was observed in the different smoking categories (Table 3).

**Table 3:** Hazard ratio (HR) and 95% confidence interval (CI) of the association of fish consumption, and risk of BC based on tertiles of intakes by gender and smoking status

	Tertile 1	Tertile 2	Tertile 3	P-trend	Tertile 1	Tertile 2	Tertile 3	P-trend
	HR (95%CI)*	HR (95%CI)	HR (95%CI)		HR (95%CI)*	HR (95%CI)	HR (95%CI)	
	Women				Men			
	Total fish and fish products				Total fish and fish products			
Case/non-case	80/44,180	94/37,374	161/34,470		172/17,309	379/23,895	651/26,459	
Person year	479,226.23	377,769.02	281,772.08		186,702.27	225,122.36	215,306.94	
Crude	1 (reference)	0.80 (0.58, 1.10)	0.60 (0.43, 0.83)	*0.001*	1 (reference)	0.75 (0.64, 0.90)	0.68 (0.58, 0.81)	< *0.001*
Model 1^a^	1 (reference)	0.77 (0.55, 1.08)	0.91 (0.61, 1.36)	*0.575*	1 (reference)	0.86 (0.72, 1.02)	0.85 (0.71, 1.02)	*0.134*
Model 2^b^	1 (reference)	0.78 (0.55, 1.09)	0.96 (0.63, 1.45)	*0.698*	1 (reference)	0.86 (0.72, 1.03)	0.81 (0.67, 0.98)	*0.036*
Model 3^c‡^	1 (reference)	1.07 (0.76, 1.51)	0.77 (0.39, 1.50)	*0.658*	1 (reference)	0.85 (0.71, 1.02)	0.79 (0.65, 0.97)	*0.047*

	HR (95%CI)*	HR (95%CI)	HR (95%CI)	P-trend	HR (95%CI)*	HR (95%CI)	HR (95%CI)	P-trend	HR (95%CI)*	HR (95%CI)	HR (95%CI)	P-trend
	Never smoker				Former smoker				Current smoker			
	Total fish and fish products				Total fish and fish products				Total fish and fish products			
Cases/non-cases	52/29,617	65/23,628	141/27,235		100/19,101	211/21,765	384/24,616		100/12,771	197/15,876	287/9078	
Person year	323,773.01	231,752.69	213,767.02		204,627.5	206,186.25	194,456.23		137,527.99	164,952.43	88,855.77	
Crude	1 (reference)	0.72 (0.49, 1.05)	0.55 (0.38, 0.80)	0.001	1 (reference)	0.83 (0.66, 1.04)	0.73 (0.58, 0.91)	0.005	1 (reference)	0.79 (0.63, 0.99)	0.78 (0.62, 0.99)	0.089
Model 1^a^	1 (reference)	0.93 (0.60, 1.43)	1.24 (0.74, 2.08)	0.434	1 (reference)	0.84 (0.66, 1.08)	0.79 (0.62, 1.01)	0.072	1 (reference)	0.87 (0.69, 1.10)	0.90 (0.71, 1.16)	0.535
Model 2^b^	1 (reference)	0.93 (0.60, 1.44)	1.24 (0.74, 2.10)	0.416	1 (reference)	0.85 (0.67, 1.09)	0.77 (0.60, 0.99)	0.042	1 (reference)	0.87 (0.69, 1.10)	0.88 (0.68, 1.13)	0.364
Model 3^c‡^	1 (reference)	0.93 (0.60, 1.43)	1.24 (0.74, 2.08)	0.592	1 (reference)	0.84 (0.66, 1.08)	1.17 (0.72, 1.95)	0.823	1 (reference)	0.87 (0.65, 1.15)	0.75 (0.44, 1.28)	0.210

*HR hazard ratio, *CI* confidence interval

^a^Adjusted for age, sex, and total energy intake

^b^Adjusted for model 1 + vegetables and fruits intakes

^c‡^Adjusted for model 2 + red meat intake

Stratified results for BC subtypes (i.e., NMIBC and MIBC) showed no different effect of any of the meat- and fish intake on the different BC subtype risks (p-heterogeneity for all > 0.05) (Supplementary Table 2).

### Dose–response and sensitivity analyses

**Fig. 1:**
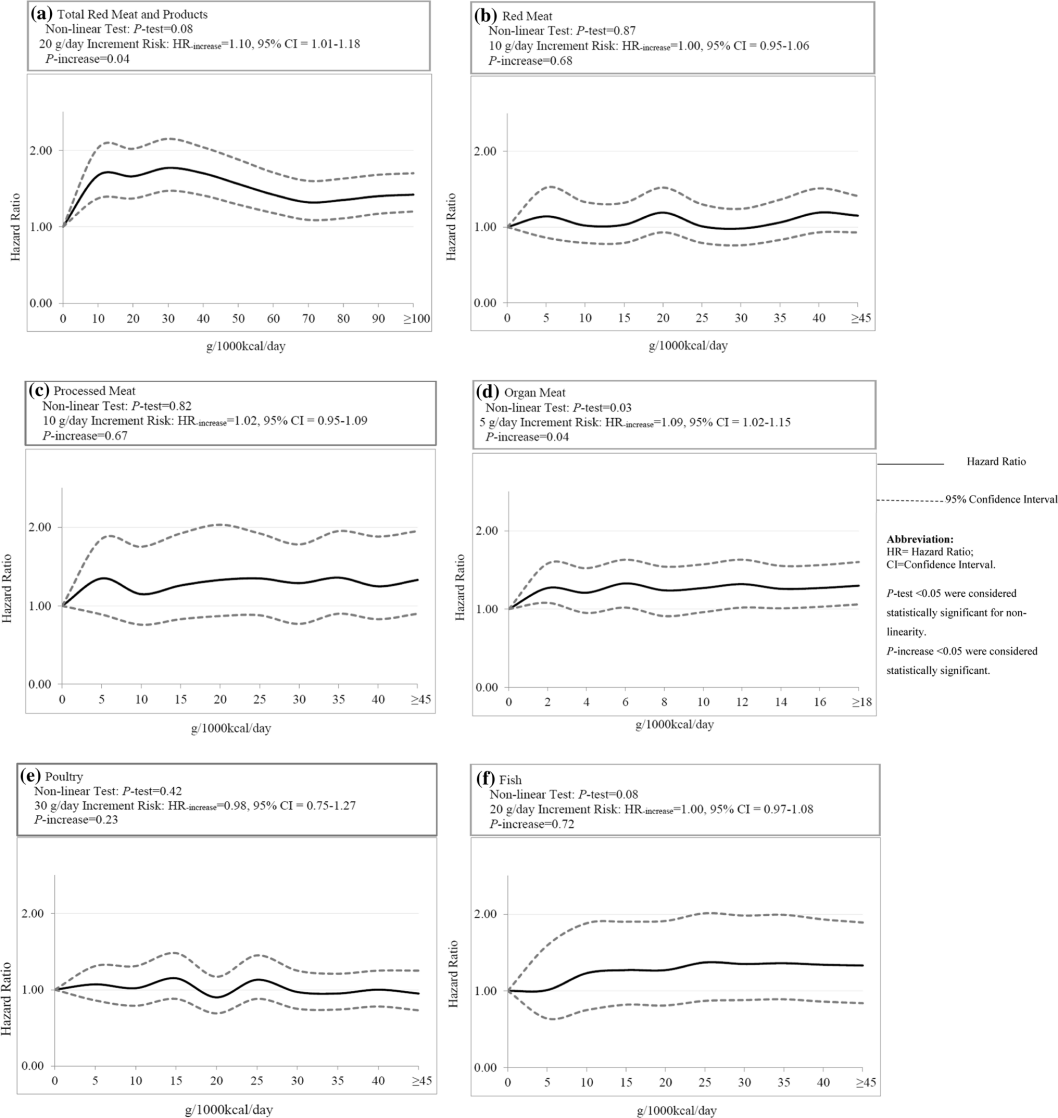
Dose–response relationships between meat intakes and the risk of bladder cancer among **a** total red meats and products; **b** red meats; **c** processed meats; **d** organ meats; **e** poultry and **f** total fish and fish products. The solid lines represent the hazard ratios (HRs); the dashed lines represent the 95% confidence intervals (CIs) for the trend. The HRs were adjusted for age (years, continuous), sex (men or women), smoking (never smokers, former smokers or current smokers), energy intake (kcal/day, continuous), vegetable intake (g/1000 kcal/day, continuous), fruit intake (g/1000 kcal/day, continuous) poultry (g/1000 kcal/day, continuous) and fish (g/1000 kcal/day, continuous) intake or red meat intake (g/1000 kcal/day, continuous) (model 3). *g* gram; *kcal* kilocalorie. Referent group was non-intake

Dose–response relationships between different sources of meat consumptions and the risk of BC are displayed in Fig. 1. Although cox-regression showed a significantly increased BC risk for organ meat consumption of over 15 g/1000 kcal/day, no significant dose–response relationship was observed for any meat-type and neither for fish (Fig. 1).

In order to determine the single study effect, sensitivity analyses were performed by removing each individual study in turn from the main analysis. Results showed that the main finding remained robust.

## Discussion

By bringing together the world’s data on meat and fish consumption and BC risk, this large prospective study demonstrates an overall significant association between high consumption of organ meat and BC risk and a slightly inverse association for high consumption of fish among men.

Epidemiological evidence on the association between organ meats and BC risk is mainly lacking. To our knowledge, only one previously conducted case-control study assessed this association [42]. In line with results from the present study, the authors found an increased BC risk among South and East Chinese individuals [42]. A possible explanation for the observed association between organ meat and BC risk, is the high fat content (especially saturated fats) of organ meat, which has been reported to increase the BC risk [43, 44]. In addition, it has been suggested that the cooking procedure of fat-rich meat forms mutagens and consequently affect BC risk [45–47]. As such, it is reported that different procedures of cooking meat i.e.; at higher temperatures (roasting) or for prolonged times (e.g. stewing), were associated with an increased BC risk [48]. Another possible explanation could be the fact that most organ meats are high in toxins [49], which might cause dysbiosis of the urinary tract, thereby indirectly causing an increased BC risk [50–52].

Bioassays and epidemiological studies indicated that tobacco smoking might modify the effect of dietary fat and cancer risk by enhancing the carcinogenic potency of meat and exerted a synergistic effect on cancer risk [53–55]. Moreover, the N-nitroso components of meat, the nitrosation of nicotine during tobacco processing, and the tobacco-specific nitrosamines resulted from cigarette smoking might lead to an increased total N-nitroso compound consumption, thereby increasing the BC risk of meat in current smokers [56]. However, in the present study no interaction between meat consumption and smoking status could be observed. This might be due residual confounding, which could not be assess in the present study.

In the present study we found no significant association between poultry intake and BC risk. This is in line with the results of a meta-analysis of eight studies, also revealing a non-significant association between poultry and BC risk (RR: 0.77, 95% CI 0.48, 1.06) [57]. However, the NIH-AARP Diet and Health study reported a statistically significant decreased BC risk associated with a 10 g/per in white meat consumption [38]. It is suggested that, compared to red meat, white meat (including poultry) contains less saturated fat and heme iron, potential inducers of oxidative stress and DNA damage [58], and release less mutagenic substitutes during the cooking procedure. It could, therefore, be possible that the previously observed inverse association between poultry and BC risk was not due to a protective effect of poultry itself, but rather due to a reduced intake of red meat, for which only limited adjustment was performed.

In the present study we found an inverse association between fish consumption and BC risk in men, but not in women. Although the evidence of the association of fish consumption and BC is scarce, a previously conducted Spanish case-control study also reported an inverse association between fish intake and BC risk [59]. This protective effect of fish on BC risk might possibly be due to the concentrated doses of anti-inflammatory, long-chain n-3 fatty acids in fish, shown to inhibit cancer development and progression [60]. On the contrary, however, several observation studies on fish intake and BC risk observed a null-association [12, 61, 62]. It is suggested that the way fish is served may be quite different between cultures and also preparation, conservation, and processing methods may have deleterious health effects (e.g. Cantonese-style salted fish or heavily battered and deep fried) [63]. So, future research is needed to elucidate the exact role of fish on the development of BC, considering also differences in fish processing.

Overall, a null-association between red- and processed meat consumption and BC risk was observed. Although this is in line with several previously conducted studies, including three cohort studies and a meta-analysis, [12–14, 57], other studies, including two meta-analyses and a cohort study, reported a direct negative association between both red- and processed meat consumption and the BC risk [15, 16, 64]. Potential mechanisms underlying the association of meat consumption and BC risk are still unclear. Therefore, future research is warranted to clarify the underlying mechanisms.

## Strengths and limitations

So far, the BLEND database is the largest pooled prospective cohort study investigating the associations between consumption of different sources of meat and the risk of developing BC and allows enough statistical power to conduct detailed analyses in detecting small effects. The use of individual participant data enables adjustments to be made for the same confounders across all studies. Additionally, eliminating possible sources of heterogeneity with the use of prospective cohort studies only, precludes recall bias which commonly occur in case-control and retrospective cohort studies.

Alternatively, several limitations to our study should be considered. Some information in the BLEND database was only in portions per week. This was converted to grams per day using the BLEND Nutrient 100-g database. However, the conversions were not country specific. Also, limited information was available for some potential risk factors of BC, such as BMI, physical inactivity, socioeconomic status, and occupational exposures to carcinogenic chemicals. The possibility to adjust for these factors will provide more accurate risk estimates. Moreover, it is a possibility that people with a high intake of fish and poultry might have generally healthier lifestyles and diets than those with a low intake, thus we could not rule out the possibility that some of the associations could be or partially due to unmeasured factors related to a healthy lifestyle than to purely white meat intakes [65]. However, the current literature suggests only a small proportion of BC cases can be attributed to lifestyle and environmental factors [66]. In addition, we were unable to take into account possible changes in dietary and lifestyle habits over time, which would better reflect the effect of long-term diet. Furthermore, it is suggested that meat might be involved in the bladder carcinogenesis via multiple potentially carcinogenic fish/meat‐related compounds related to cooking and processing, including nitrate, nitrite, HCAs, and PAHs. However, in this study there was no information on meat preparation or cooking methods. Besides, for most cohorts, the exposure variable was assessed by FFQs, therefore, measurement error and misclassification of study participants in terms of the exposure and outcome are unavoidable. Likewise, information bias, as a consequence of self-reported information on food consumption is a common bias in nutritional studies [67]. However, the strength and direction of this bias should not be significantly different between cases and non-cases, suggesting that the impact of information bias on our findings might be minimal. Finally, the present study sample consisted mostly of Caucasians, and this may limit the generalizability of our results to other racial/ethnic populations or geographic regions.

## Conclusion

In summary, this large prospective study added new insights into the role of meat consumptions toward BC carcinogenesis. It was found that organ meats may be a risk factor for the development of BC, and fish might play a protective role against BC risk among men.

## Supplementary Information

Below is the link to the electronic supplementary material.

Supplementary file1 (DOCX 23 kb)

## Data Availability

Datasets that are minimally required to replicate the outcomes of the study will be made available upon reasonable request.

## Abbreviations

BC: Bladder cancer
BLEND: Bladder cancer Epidemiology and Nutritional Determinants
BMI: Body Mass Index
CI: Confidence Interval
EPIC: European Prospective Investigation into Cancer
FFQ: Food Frequency Questionnaire
g: Gram
HRs: Hazard Ratios
HCAs: Heterocyclic Amines
ICD-O: International Classification of Diseases for Oncology
kcal: Kilocalorie
ml: Milliliter
MIBC: Muscle-invasive BC
NOC: N-nitroso Compounds
NMIBC: Non-muscle-invasive BC
NLCS: The Netherlands Cohort Study
PLCO: The Prostate, Lung, Colorectal, and Ovarian cohort study
PAHs: Polycyclic aromatic hydrocarbons
RR: Relative Risk
SD: Standard Deviation
VITAL: VITamins and Lifestyle study
WCRF: World Cancer Research Fund International
