# Supplementary material for: The association between meat and fish consumption and bladder cancer risk: a pooled analysis of 11 cohort studies
Source: Eur J Epidemiol. 2021 May 25;36(8):781–92. doi: 10.1007/s10654-021-00762-4 (PMC8416827; doi:10.1007/s10654-021-00762-4)
Supplement: Supplementary file 1 — Supplementary file1 (DOCX 23 kb) [file 10654_2021_762_MOESM1_ESM.docx]

**Supplementary Tables**

**Supplementary Table 1.** Additional baseline characteristic for included studies in BLEND study.

| Characteristics | NLCS | VITAL | EPIC-Denmark | EPIC-France | EPIC-Germany | EPIC-Italy | EPIC-Spain | EPIC-Sweden | EPIC-the Netherlands | EPIC- the UK | EPIC-Norway |
| --- | --- | --- | --- | --- | --- | --- | --- | --- | --- | --- | --- |
|  | N=5,238 | N=66,518 | N=55,670 | N=64,204 | N=48,754 | N= 44,663 | N=40,389 | N=48,625 | N=36,801 | N= 74,379 | N=33,304 |
| Initiate year of baseline assessment | 1986 | 2000 | 1993 | 1990 | 1994 | 1992 | 1992 | 1991 | 1993 | 1993 | 1991 |
| Length of follow up (years) | 14 | 7 | 11 | 10 | 10 | 11 | 12 | 13 | 12 | 11 | 10 |
| Subjects (number) |  |  |  |  |  |  |  |  |  |  |  |
| Case/ | 876/ | 337/ | 386/ | 31/ | 205/ | 186/ | 149/ | 301/ | 107/ | 247/ | 23/ |
| non-case | 4.362 | 66.181 | 55.284 | 64.173 | 48.549 | 44.477 | 40.24 | 48.324 | 36.694 | 74.132 | 33.281 |
| Person-year | 73,688.8 | 448,995.4 | 608.813 | 667,809.9 | 482,453.3 | 502,020.3 | 487,491.1 | 638,482.8 | 434,974.5 | 828,991.7 | 6,437,305.7 |
| Baseline age (years) (mean ±SD) |  |  |  |  |  |  |  |  |  |  |  |
| Case | 62.73 (4.09) | 66.16 (7.01) | 58.50 (4.37) | 58.04 (6.00) | 56.41 (7.13) | 55.24 (6.75) | 54.49 (7.19) | 60.27 (7.07) | 56.20 (8.03) | 63.62 (9.98) | 49.30 (4.38) |
| Non-case | 61.85 (4.21) | 61.18 (7.37) | 56.67 (4.37) | 52.74 (6.63) | 50.55 (8.56) | 50.50 (7.92) | 49.19 (8.03) | 51.93 (10.89) | 48.94 (11.93) | 49.05 (14.34) | 48.07 (4.30) |
| Sex n (%) |  |  |  |  |  |  |  |  |  |  |  |
| Men | 2,867 (54.73) | 33,394 (50.20) | 26,532 (47.66) | 0 (0.00) | 21,168 (43.42) | 13,774 (30.84) | 15,259 (37.78) | 22,214 (45.68) | 9,629 (26.17) | 22,260 (29.93) | 0 (0.00) |
| Women | 2,371 (45.27) | 33,124 (49.80) | 29,138 (52.34) | 64,204 (100.00) | 27,586 (56.58) | 30,889 (69.16) | 25,130 (62.22) | 26,411 (54.32) | 27,172 (73.83) | 52,119 (70.07) | 33,304 (100.00) |

*Abbreviations:*

EPIC: European Prospective Investigation into Cancer

NLCS: The Netherlands Cohort Study

VITAL: VITamins and Lifestyle study

**Supplementary Table 2.** Hazard ratio (HR) and 95% confidence interval (CI) of the association of meat and meat types, and risk of BC based on tertiles of meat and meat types for stratification of bladder cancer subtype.

|  | Tertile 1 | Tertile 2 | Tertile 3 | P trend |  | Tertile 1 | Tertile 2 | Tertile 3 | P trend |
| --- | --- | --- | --- | --- | --- | --- | --- | --- | --- |
|  | **HR (95%CI) *** | **HR (95%CI)** | **HR (95%CI)** |  |  | **HR (95%CI) *** | **HR (95%CI)** | **HR (95%CI)** |  |
| NMIBC | | | |  | **MIBC** | | | |  |
| *Total red meat and products* | | | |  | ***Total red meat and products*** | | | |  |
| Person year | 2581.20 | 3185.39 | 2392.94 |  | Person year | 1628.40 | 2632.94 | 1242.26 |  |
| Crude | 1 (reference) | 1.20 (1.05, 1.38) | 1.13 (0.99, 1.30) | *0.092* | Crude | 1 (reference) | 1.23 (1.04, 1.45) | 1.23 (1.04, 1.45) | *0.018* |
| Model 1 ^1^ | 1 (reference) | 1.17 (1.01, 1.36) | 1.17 (1.01, 1.36) | *0.045* | Model 1 ^1^ | 1 (reference) | 1.04 (0.86, 1.25) | 1.03 (0.85, 1.24) | *0.760* |
| Model 2 ^2^ | 1 (reference) | 1.27 (1.09, 1.48) | 1.26 (1.09, 1.47) | *0.004* | Model 2 ^2^ | 1 (reference) | 1.18 (0.98, 1.43) | 1.22 (1.00, 1.49) | *0.049* |
| Model 3 ^3†^ | 1 (reference) | 0.94 (0.75, 1.17) | 1.23 (0.92, 1.64) | *0.302* | Model 3 ^3†^ | 1 (reference) | 0.98 (0.77, 1.25) | 1.18 (0.82, 1.68) | *0.522* |
| *Red Meats* | | | |  | ***Red Meats*** | | | |  |
| Person year | 3035.28 | 3028.05 | 2078.18 |  | Person year | 2006.56 | 2550.43 | 917.253 |  |
| Crude | 1 (reference) | 0.81 (0.62, 1.07) | 0.75 (0.57, 0.98) | *0.047* | Crude | 1 (reference) | 0.79 (0.40, 1.54) | 0.77 (0.39, 1.53) | *0.611* |
| Model 1 ^1^ | 1 (reference) | 0.59 (0.45, 0.79) | 0.47 (0.35, 0.62) | *<0.001* | Model 1 ^1^ | 1 (reference) | 0.40 (0.20, 0.82) | 0.40 (0.19, 0.82) | *0.155* |
| Model 2 ^2^ | 1 (reference) | 0.61 (0.46, 0.81) | 0.52 (0.39, 0.69) | *<0.001* | Model 2 ^2^ | 1 (reference) | 0.39 (0.19, 0.81) | 0.43 (0.21, 1.02) | *0.299* |
| Model 3 ^3†^ | 1 (reference) | 1.00 (0.81, 1.25) | 1.16 (0.89, 1.49) | *0.457* | Model 3 ^3†^ | 1 (reference) | 0.94 (0.74, 1.20) | 1.06 (0.79, 1.41) | *0.381* |
| *Processed Meats* | | | |  | ***Processed Meats*** | | | |  |
| Person year | 1364.27 | 1235.78 | 1110.98 |  | Person year | 352.62 | 387.88 | 229.48 |  |
| Crude | 1 (reference) | 1.23 (1.02, 1.49) | 1.25 (1.03, 1.52) | *0.021* | Crude | 1 (reference) | 1.14 (0.85, 1.54) | 1.63 (1.18, 2.26) | *0.005* |
| Model 1 ^1^ | 1 (reference) | 1.34 (1.09, 1.65) | 1.35 (1.09, 1.66) | *0.005* | Model 1 ^1^ | 1 (reference) | 1.46 (1.04, 2.06) | 1.65 (1.16, 2.34) | *0.004* |
| Model 2 ^2^ | 1 (reference) | 1.36 (1.10, 1.68) | 1.40 (1.13, 1.73) | *0.002* | Model 2 ^2^ | 1 (reference) | 1.47 (1.04, 2.08) | 1.82 (1.28, 2.60) | *0.001* |
| Model 3 ^3†^ | 1 (reference) | 2.11 (1.18, 3.77) | 1.09 (0.48, 2.45) | *0.972* | Model 3 ^3†^ | 1 (reference) | 1.48 (1.05, 2.11) | 1.91 (1.32, 2.72) | *0.001* |
| *Organ Meats* | | |  |  | ***Organ Meats*** | | | |  |
| Person year | 543.42 | 1641.58 | 1526.03 |  | Person year | 109.86 | 432.60 | 427.52 |  |
| Crude | 1 (reference) | 1.04 (0.78, 1.37) | 0.97 (0.72, 1.29) | *0.688* | Crude | 1 (reference) | 1.46 (0.75, 2.83) | 1.19 (0.59, 2.39) | *0.917* |
| Model 1 ^1^ | 1 (reference) | 1.43 (1.07, 1.90) | 1.22 (0.91, 1.64) | *0.417* | Model 1 ^1^ | 1 (reference) | 1.21 (062, 2.36) | 1.08 (0.52, 2.26) | *0.951* |
| Model 2 ^2^ | 1 (reference) | 1.45 (1.07, 1.95) | 1.27 (0.93, 1.72) | *0.351* | Model 2 ^2^ | 1 (reference) | 1.03 (0.47, 2.25) | 0.94 (0.43, 2.08) | *0.758* |
| Model 3 ^3†^ | 1 (reference) | 1.46 (1.08, 1.96) | 1.26 (0.92, 1.70) | *0.280* | Model 3 ^3†^ | 1 (reference) | 1.07 (0.51, 2.31) | 0.96 (0.47, 2.12) | *0.321* |
| *Poultry* | | |  |  | ***Poultry*** | | | |  |
| Person year | 3383.46 | 2907.79 | 1868.28 |  | Person year | 2477.57 | 2062.90 | 963.12 |  |
| Crude | 1 (reference) | 1.08 (0.94, 1.25) | 0.98 (0.85,1.12) | *0.700* | Crude | 1 (reference) | 0.89 (0.74, 1.06) | 0.81 (0.68, 0.97) | *0.019* |
| Model 1 ^1^ | 1 (reference) | 1.03 (0.89, 1.20) | 0.83 (0.71, 0.97) | *0.023* | Model 1 ^1^ | 1 (reference) | 0.72 (0.60, 0.88) | 0.75 (0.62, 0.91) | *0.004* |
| Model 2 ^2^ | 1 (reference) | 1.00 (0.86, 1.16) | 0.85 (0.73, 0.99) | *0.046* | Model 2 ^2^ | 1 (reference) | 0.80 (0.66, 0.97) | 0.80 (0.66, 0.98) | *0.028* |
| Model 3 ^3‡^ | 1 (reference) | 1.35 (1.06, 1.70) | 1.27 (0.97, 1.67) | *0.047* | Model 3 ^3‡^ | 1 (reference) | 1.20 (0.74, 1.94) | 1.03 (0.57, 1.84) | *0.447* |
| *Total fish and fish products* | | | |  | ***Total fish and fish products*** | | | |  |
| Person year | 556.97 | 1407.47 | 2717.96 |  | Person year | 168.77 | 1136.48 | 2539.23 |  |
| Crude | 1 (reference) | 0.92 (0.69, 1.23) | 0.65 (0.49, 0.85) | *<0.001* | Crude | 1 (reference) | 0.59 (0.39, 0.89) | 0.48 (0.33, 0.71) | *<0.001* |
| Model 1 ^1^ | 1 (reference) | 0.89 (0.66, 1.21) | 0.61 (0.45, 0.82) | *<0.001* | Model 1 ^1^ | 1 (reference) | 0.56 (0.36, 0.87) | 0.42 (0.28, 0.63) | *<0.001* |
| Model 2 ^2^ | 1 (reference) | 0.93 (0.68, 1.25) | 0.70 (0.51, 0.94) | *0.001* | Model 2 ^2^ | 1 (reference) | 0.58 (0.37, 0.90) | 0.47 (0.31, 0.72) | *0.001* |
| Model 3 ^3‡^ | 1 (reference) | 1.04 (0.57, 1.14) | 0.81 (0.42, 0.85) | *0.001* | Model 3 ^3‡^ | 1 (reference) | 0.54 (0.34, 0.93) | 0.46 (0.29, 0.73) | *0.001* |

^*^HR=Hazard Ratio, CI=confidence interval.

^1^ adjusted for age, sex, smoking status and total energy intake.

**^2^** adjusted for model 1 + vegetables and fruits intakes.

^3^ adjusted for †model 2+ poultry and fish intake ‡model 2 + red meat intake.
